# Subnational introduction of the RTS,S/AS01_E_ malaria vaccine into routine immunization: experience and lessons from the three pilot countries

**DOI:** 10.1186/s12936-025-05484-6

**Published:** 2025-07-28

**Authors:** Rose Jalang’o, Kwame Amponsa-Achiano, Mike Chisema, Keziah Malm, Lydiah Khalayi, Brenda Mhone, Wahjib Mohammed, Franklin Asiedu-Bekoe, Adam Haji, Josephine Njoroge, Boston Zimba, Esther Chirwa, Peter O. Tweneboah, Jackson Sillah, Mgaywa G. M. D. Magafu, Cynthia Bergstrom, Tracey Goodman, Jenny Walldorf, Kristen Kelleher, Eliane Pellaux-Furrer, Mary J. Hamel, Michael R. Adjei, Rafiq N. A. Okine

**Affiliations:** 1 National Vaccines and Immunization Programme, Nairobi, Kenya; 2 Expanded Programme on Immunization, Public Health Division, Ghana Health Service, Accra, Ghana; 3 Ministry of Health, Lilongwe, Malawi; 4 National Malaria Elimination Programme, Public Health Division, Ghana Health Service, Accra, Ghana; 5 Public Health Division, Ghana Health Service, Accra, Ghana; 6 World Health Organization Country Office, Nairobi, Kenya; 7 World Health Organization Country Office, Lilongwe, Malawi; 8 World Health Organization Country Office, Accra, Ghana; 9 Tropical and Vector-Borne Diseases Unit, World Health Organization Africa Regional Office, Brazzaville, Congo; 10 Vaccine-Preventable Diseases Unit, World Health Organization Africa Regional Office, Brazzaville, Congo; 11 Department of Immunization, Vaccines, and Biologicals, World Health Organization, Geneva, Switzerland; 12 Global Malaria Programme, World Health Organization, Geneva, Switzerland

**Keywords:** Malaria vaccine, RTS,S/AS01, Malaria Vaccine Implementation Programme, New vaccine introduction, Pilot implementation, Ghana, Kenya, Malawi, Malaria prevention, Malaria vaccine coverage

## Abstract

**Background:**

In October 2021, the World Health Organization (WHO) recommended the RTS,S/AS01_E_ (RTS,S) malaria vaccine for the prevention of *Plasmodium falciparum* malaria in children living in endemic areas informed by evidence from the subnational pilot introduction and evaluation in Ghana, Kenya, and Malawi as part of the WHO-coordinated Malaria Vaccine Implementation Programme (MVIP). With the global vaccine supply boosted by the pre-qualification of a second malaria vaccine, R21/Matrix-M (R21), in October 2023, many endemic countries (20 as of April 2025) have introduced malaria vaccines into their national childhood immunization and malaria control programmes. More endemic countries are expected to introduce or scale up malaria vaccines in 2025 and beyond. This paper summarizes key operational lessons from the pilot countries to facilitate the introduction and scale-up of malaria vaccination in other countries.

**Methods:**

Pilot areas were identified, in part, based on local malaria epidemiology. RTS,S was initially introduced in randomly selected areas, while other areas served as comparators until the four-dose schedule vaccine was scaled up following the WHO recommendation in 2021. In Ghana and Kenya, the vaccine was administered at ages 6, 7, 9, and 24 months (Ghana switched to administer the fourth dose at age 18 months in 2023), and Malawi chose a schedule of 5, 6, 7, and 22 months.

**Results:**

Vaccination coverage improved over time, reaching about 80% for the first dose and around 75% for the third dose by 2023 in the initial pilot areas. Implementation challenges included an inadequate understanding of age eligibility among healthcare workers during the early phase of introduction, low fourth dose coverage (with a median coverage of 46% in 2023 across the three countries), and disruptions to service delivery caused by disease outbreaks and other natural disasters. Health stakeholders and caregivers attested to the positive impact of introducing the malaria vaccine, including a reduction in malaria hospitalizations and the strengthening of the National Immunization Programme (NIP) through routine immunization refresher training and supportive supervision.

**Conclusions:**

The pilot highlighted lessons for malaria vaccine introduction: (1) clearly outlined roles and responsibilities of key stakeholders including NIP and National Malaria Programme (NMP); (2) appropriate approach to vaccine introduction launch, communication, and demand generation to enhance vaccine uptake; (3) flexibility with dose scheduling to optimize coverage; and (4) updated data collection tools for accurate documentation, and data quality.

**Supplementary Information:**

The online version contains supplementary material available at 10.1186/s12936-025-05484-6.

## Background

Globally, malaria cases have increased from an estimated 244 million in 2021 to 249 million in 2022, with approximately 94% of cases recorded in the World Health Organization (WHO) African Region [1]. Malaria-related mortality rates declined by approximately 31% between 2000 and 2023, with a nearly 29% decrease occurring in the African Region during the same period. Despite the considerable progress, mortality among children under 5 years in the African Region accounted for 76% of the global malaria-related deaths [1].

Challenges to further progress include biological threats, such as the emergence of insecticide-resistant mosquito species and drug-resistant *Plasmodium* strains, which may compromise the effectiveness of tools like insecticide-treated nets and antimalarial drugs. A new tool, the malaria vaccine, has been shown to reduce malaria morbidity and mortality among children [2].

Following the European Medicines Agency's (EMA) positive scientific opinion on the malaria vaccine, RTS,S/AS01_E_ (RTS,S) in 2015 [3], the WHO recommended large-scale pilot implementation of the four-dose regimen in children aged at least 5 months at the first dose, in settings with moderate to high malaria transmission. The Malaria Vaccine Implementation Programme (MVIP) was designed to assess the feasibility of implementing four doses of the vaccine in a schedule of the National Immunization Programme (NIP), evaluate safety, and measure the impact of vaccine introduction in the context of routine use [4].

After a call for expressions of interest by the WHO, Ghana, Kenya, and Malawi were selected to participate in the pilot introductions in 2017. In surveys conducted before the pilot implementation, malaria parasite prevalence among children under five years of age in the initial implementing areas was 21%, 26%, and 28% in Ghana, Kenya, and Malawi, respectively [2]. The proportion of deaths attributed to malaria among this age group was estimated at 34% in Ghana [5], 33.2% in Kenya [6], and 20% in Malawi [7].

The Ministries of Health (MoHs) first introduced the RTS,S vaccine in selected subnational areas using routine systems in 2019. The COVID-19 pandemic disrupted healthcare worker refresher training, supportive supervision, and service delivery in the countries. Nonetheless, the outcome and evidence from the pilot were positive. In October 2021, the WHO recommended the broader use of the RTS,S malaria vaccine for the prevention of *Plasmodium falciparum* malaria in children living in endemic areas [8]. With the global vaccine supply boosted by the pre-qualification of a second malaria vaccine, R21/Matrix-M (R21), in 2023, at least 26 countries are set to introduce the malaria vaccine into childhood immunization and national malaria programmes (NMPs) from 2024 [9].

The lessons and experiences accrued in the MVIP should be a valuable resource for new countries planning introductions or scale-ups, irrespective of the malaria vaccine product type. This paper summarizes key operational lessons from the pilot countries to facilitate the implementation of malaria vaccines in other countries.

## Planning, implementation, lessons, and challenges of the MVIP

Key lessons documented during implementation reinforce the importance of strengthening the healthcare system and collaborating with communities to enhance vaccine uptake and impact [10]. During the pilot implementation, challenges, best practices, and lessons were documented in various ways. For example, the countries' documented healthcare worker experience and feedback from the supportive supervisory visits and performance review meetings. The WHO also facilitated quarterly cross-country peer learning meetings to share best practices and develop strategies to address common challenges. The feedback on gaps and implementation challenges was also documented using standardized checklists at various points during the pilot implementation. The lessons were also collated from the various country presentations during the pilot. These specific lessons and experiences accrued during the pilot introduction of the malaria vaccine are presented in this paper.

### Country decision-making process and selection criteria for implementing areas

The selection criteria for the pilot countries included demonstrated engagement and interest from MoHs; presence of functional immunization and malaria programmes as evidenced by the coverages of third dose of diphtheria-tetanus-pertussis vaccine (DTP3/Penta3) and first dose of measles-rubella vaccine (MR1), insecticide-treated net (ITN) usage; high malaria burden; presence of at least one sentinel hospital per region to facilitate the collection of quality safety data on meningitis and cerebral malaria; and national pharmacovigilance readiness. Experience in the RTS,S Phase 3 clinical trial was also considered favourable [4].

The MoHs decided to participate in the MVIP following the recommendations of the National Immunization Technical Advisory Groups (NITAGs). Key factors, including cost, financing, and health impact, were taken into consideration. Given the burden of malaria in the respective countries, the decision to participate in the MVIP was strongly supported by in-country stakeholders, who weighed the benefits against anticipated costs. The MoHs expressed interest in collaborating on the MVIP, and in April 2017, the WHO announced the selection of the three countries for the pilot programme.

The vaccine was introduced in subnational areas based on selection criteria including malaria burden, intensity of transmission (malaria parasite prevalence > 20%), and immunization programme performance.

### Coordination and formation of technical working groups

The early formation of technical working groups (TWGs) and subcommittees was crucial in facilitating the implementation process. TWGs or equivalents were constituted in each country to oversee the planning and implementation of the MVIP. In Ghana, the formation of the TWG predated the call for expression of interest in piloting the malaria vaccine [11]. The TWGs comprised representatives from state and non-state agencies, including the NIP, national malaria programme (NMP), national regulatory authority (NRA), WHO, and other global immunization and malaria partners, civil society organizations (CSOs), and media organizations.

TWG sub-committees were assigned specific tasks, including advocacy, communication, social mobilization, pharmacovigilance, monitoring and evaluation, training, and resource mobilization. The national-level coordination mechanisms were replicated at the subnational levels in Ghana to foster ownership and facilitate programme implementation.

**Fig. 1:**
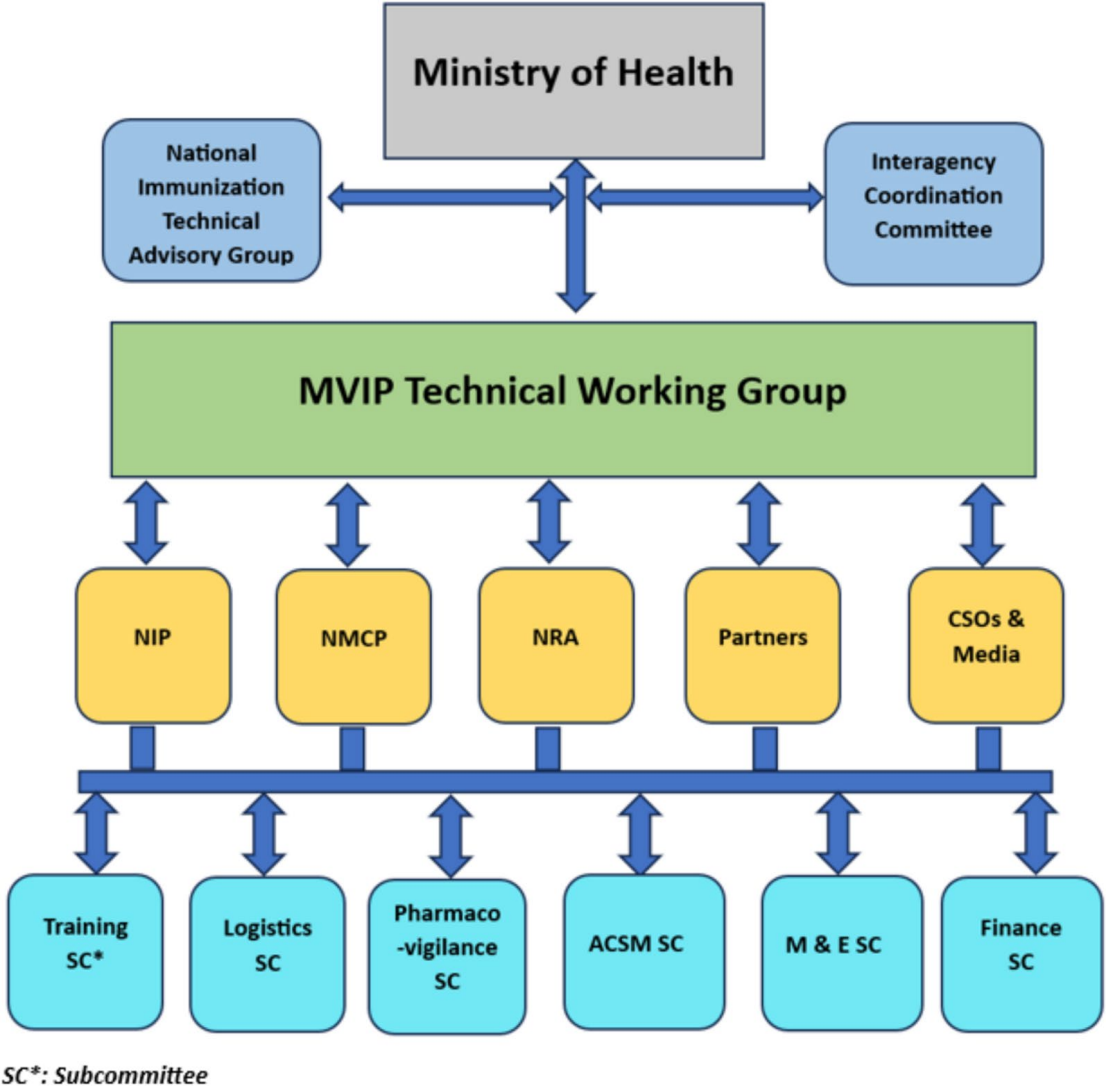
Generic structure of country coordination mechanism, MVIP; 2019–2023. *ACSM* Advocacy, Communication, and Social Mobilization, *CSO* Civil Society Organization, *NIP* National Immunization Programme, *NMCP* National Malaria Control Programme, *NRA* National Regulatory Authority, *M&E* Monitoring and Evaluation, *SC* Subcommittee

Figure 1 illustrates the generic structure of coordination mechanisms across the three countries.

Comprehensive national malaria vaccine introduction plans were developed, incorporating multi-sectoral engagement platforms, risk communication strategies, and community engagement approaches. Close collaboration was established and maintained between the national immunization and malaria programmes and with components of the health system responsible for information management and community health through the TWGs. The malaria programme provided epidemiological data to identify and prioritize areas for implementation, supported the development of training modules, facilitated capacity building, and participated in joint supportive supervision activities during the initial stages of vaccine introduction.

The TWG meetings provided regular touchpoints for sharing information, devising solutions to barriers and challenges, and keeping partners and stakeholders informed of progress. In Kenya, the MoH collaborated with the Pharmacy and Poisons Board (PPB) to authorize the RTS,S malaria vaccine for the pilot. The TWG in Ghana supported the NITAG in gathering and evaluating evidence, facilitating the recommendation of the MVIP for approval by the MoH. In Malawi, the malaria vaccine introduction plan was jointly endorsed by the TWG and NITAG before adoption by the MoH.

After the first year of the pilot, the membership and functionality of the TWGs evolved. The NIP and NMP technical officers who were members continued as focal points, with the NITAGs providing technical guidance for the pilots. Communication was maintained with the TWG members via emails and WhatsApp group messaging to share relevant information. Ad hoc meetings were organized to address specific issues, and when the support of collaborating agencies was required to implement specific activities, such as joint monitoring and supervision, advocacy, communication, and social mobilization (ACSM).

### Overview of subnational implementation

**Fig. 2:**
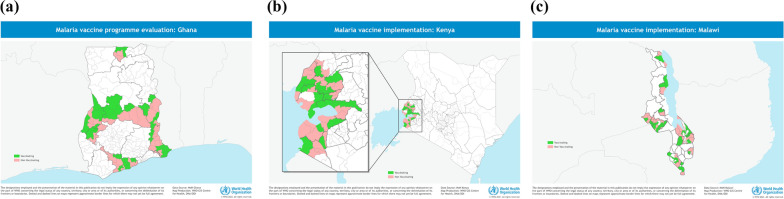
**A**–**C** Implementing subnational areas, MVIP, Ghana (**A**), Kenya (**B**), and Malawi (**C**); 2019–2023

In Ghana, the RTS,S malaria vaccine was initially introduced in 42 districts (hereafter referred to as “initial implementing areas”) across seven regions following the national launch on 30 April 2019. On 20 February 2023, the introduction of the vaccine was expanded to the remaining 51 comparator districts (hereafter referred to as “expansion areas”) of the MVIP (Fig. 2A).

Kenya selected eight counties from the lake endemic region, where malaria prevalence was high compared with other parts of the country [12] (Fig. 2B). It introduced the vaccine in 26 sub-counties after the MVIP launch on 20 September 2019. The introduction was expanded to the 25 comparator sub-counties from 7 March 2023. In Malawi, the MVIP activities were implemented in 46 clusters selected from 11 districts (Fig. 2C). Vaccination began in the 23 initial clusters following the national launch on April 23, 2019, and was expanded to the comparator clusters on November 27, 2022. The introduction of the malaria vaccine in the expansion areas occurred following the WHO's recommendation in October 2021.

Although high-profile launches were implemented in both Ghana and Kenya, a “silent” launch was implemented in Malawi. The high-profile launches were associated with higher community awareness and initially higher coverage that stabilized thereafter, but possibly contributed to some initial anti-vaccine sentiments. It is not unusual for the introduction of a new vaccine to generate anti-vaccine activity, and this was the first public health administration of a malaria vaccine to young children. Malawi’s approach, on the other hand, was characterized by lower community awareness and slower uptake, which increased over time following intensified community engagement in the pilot areas; however, there was less anti-vaccine agitations Although critical assessment of local context is essential in determining the appropriate approach, early and continuous community engagements are key in creating awareness and generating demand among caregivers to improve vaccine uptake.

Extensive stakeholder engagement, including with the media, took place at both the national and sub-national levels, preceding the introduction of vaccines in all pilot countries'initial and expansion areas. In Ghana, separate engagements were held with the parliamentary select committee on health, traditional leaders (chiefs and queen mothers), and the Ghana Academy of Arts and Sciences (GAS) to mobilize support for the MVIP. County political leaders, religious leaders, and the Paediatric Association were engaged in Kenya to strengthen demand for vaccination. Malawi held advocacy meetings with the Paediatric Association and the Traditional Healers’ group to gain support. Healthcare workers and community health educators sensitized caregivers using local channels, such as community information centres (CICs) in Ghana. Social and religious gatherings were also leveraged to generate demand for malaria vaccination. These influential stakeholders played a crucial role in motivating vaccine uptake, addressing questions and misinformation about the malaria vaccine, and fostering trust in the malaria vaccination programmes.

Measures were instituted to minimize the influx of children from non-vaccinating areas to vaccinating areas to receive the malaria vaccine during the initial phase of the pilot. Although only age-eligible children living and accessing immunization services from the vaccinating areas qualified for malaria vaccination, healthcare workers in Kenya communicated with community leaders in border communities to limit the influx, which was less than 10% [13]. Similar measures were implemented in Ghana and Malawi. However, in all countries, age-eligible children who presented at service delivery points were vaccinated, irrespective of their place of residence.

### Healthcare workers'capacity building and service delivery

In all three countries, training-of-trainers (ToT) workshops were organized to build the capacity of national and subnational teams to facilitate the training of frontline health staff. This approach ensured the availability of skilled staff to transfer knowledge to peers in the implementing areas after the structured training. To maintain the quality of the training and minimize information loss due to the cascade training approach, staff from national and regional levels were assigned to support training at the health facility level. The rapid post-introduction assessment also provided an opportunity to reinforce key messages to healthcare workers.

The countries adapted generic WHO training modules, which included content on vaccine safety and effectiveness, vaccine eligibility and administration, cold chain management and monitoring, demand generation, risk communication, and community engagement. Additional details on healthcare worker training from a qualitative study are also presented in this collection [13]. The WHO essential training packages for malaria vaccine introduction, which include lessons learned from pilot country experiences, are available to countries planning the introduction of malaria vaccines [14].

In Malawi and Ghana, capacity building and support for healthcare workers continued, with updates provided through social media platforms such as WhatsApp messaging, to ensure that comprehensive health messaging on malaria vaccines was understood and conveyed to caregivers during clinic visits. To facilitate healthcare worker decision-making on eligibility, Ghana and Malawi developed job aids (Supplement Fig. S1), interactive quizzes (https://form.typeform.com/to/gEFKoniS?typeform-source=ndean.typeform.com) [15], and videos (https://www.dropbox.com/sh/hutixxn0o48paxk/AACbei8T_BeCDDofrBfVx-eMa?dl=0&preview=rtss+eligibility+ghana+2023+HD+00.mp4) [16] on eligibility criteria that included vaccination scenarios and were based on tools adapted from the WHO. These initiatives were considered instrumental in strengthening knowledge and practice on malaria vaccines through remote means, especially during the COVID-19 pandemic, when limited mobility adversely affected opportunities for in-person refresher training and supportive supervisory visits. WHO and PATH supported the development and implementation of these innovations.

### Schedule choice for countries and rationale

**Fig. 3:**
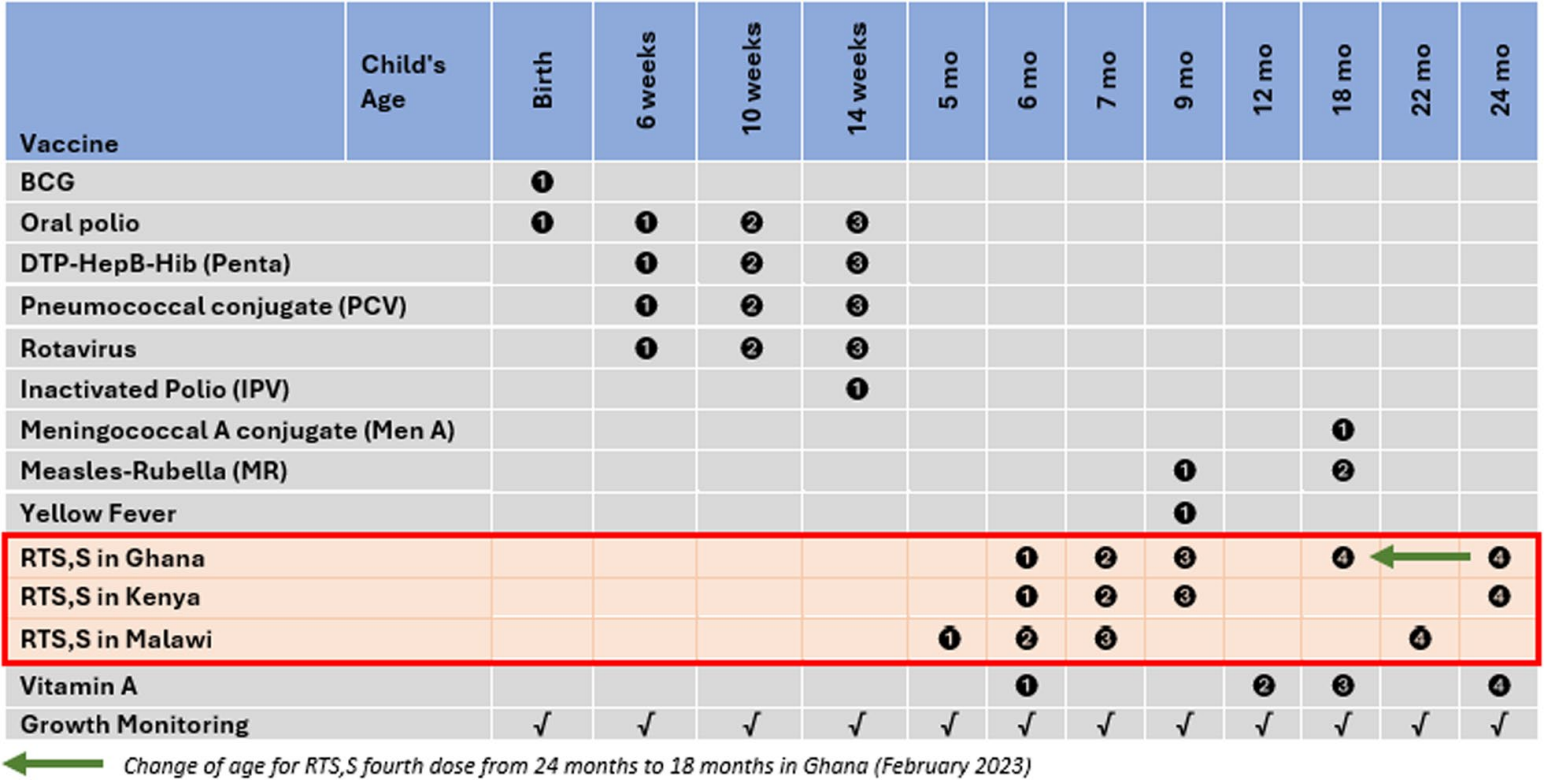
RTS,S malaria vaccine schedules for pilot countries (*inserted in the childhood immunization schedule for Ghana as of December 2023*), MVIP; 2019–2023; In February 2023, Ghana shifted the age for the RTS,S fourth dose from 24 to 18 months to align with the scheduled age for MR2 and MenA

The pilot countries selected vaccination schedules based on the feasibility of delivery in routine systems and the convenience of caregivers regarding adherence to immunization clinic appointments to complete full vaccination. The schedules of the respective doses were aligned with existing antigens and interventions in the child health package as much as possible to improve the efficiency of service delivery, while also considering the age of children with a high malaria burden. This was in line with the policy recommendation of the WHO regarding flexibility of schedule choice, including aligning with existing child health interventions and national catch-up policies. The minimum interval between the first three doses was 4 weeks, and between doses 3 and 4 was 15–18 months (a recommendation by the WHO that has since been revised to 6–18 months). The first three doses were administered from 5 months of age in Malawi, using a 5, 6, and 7-month schedule. Additionally, the first three doses were administered from 6 months of age in Ghana and Kenya, with a 6-, 7-, and 9-month schedule. The fourth dose was administered at 22 months of age in Malawi and 24 months in Ghana and Kenya (Fig. 3).

All three countries adopted an expanded age approach for the first dose at introduction, though these were implemented differently. In Kenya, children aged 6–11 months were eligible for the first dose from the first month of introduction. This was actively communicated to healthcare workers and caregivers. Ghana adopted an expanded age range of 6 −7 months for dose 1 in the first month of introduction and gradually eased eligibility to 6–11 months of age for dose 1 in subsequent months (Fig. S1). In Malawi, children aged 5–11 months were eligible for the first dose, although this was not actively enforced. The countries tailored the age eligibility to local contexts, including projections based on the availability of vaccines. It is, however, essential to note that the expanded age approach did not result in vaccine stock-outs. In Kenya and Ghana, coverage levels stabilized after comparatively high uptake in the first few months. The choice of an expanded approach also provided the opportunity to reach a critical proportion of the population at risk at the time of introduction. It facilitated the screening for age eligibility by healthcare workers to reduce missed opportunities for vaccination, particularly for children who presented late. This valuable lesson on the use of an expanded age approach for the first dose has been adopted by several countries in the recent malaria vaccine introductions.

The introduction of the malaria vaccine was used to evaluate the feasibility and acceptability of new immunization touchpoints, including visits beyond the second year of life (2YL). In all three countries, a malaria vaccine schedule was introduced at 6 months of age to strengthen the uptake of vitamin A. Independent household surveys conducted at three time points during the pilot program showed a marginal increase in Vitamin A coverage (as measured in the 6 months preceding each survey), with no significant differences in implementation and comparison (expansion) areas by 2022 [V. Mwapasa, pers. commun,19 June 2025]. Thus, the introduction of the malaria vaccine at 6 months of age did not directly increase Vitamin A uptake; however, it provides a good opportunity for countries to screen and optimize the delivery of Vitamin A supplementation and other child health services.

Because some of the dose schedules were not aligned with existing interventions in the child health package, caregivers needed additional visits to complete all four doses. High coverage of the first three doses was achieved in all three countries, although initially at variable rates. While some caregivers forgot appointment dates due to competing demands, others were unclear about when to bring their children for subsequent vaccinations because of an inadequate understanding of the schedule [17–19]. Rumours, previous negative experiences with immunization, and the burden of accessing service delivery points contributed to caregivers initially delaying uptake.

Over time, concerns about adverse events diminished, and the anticipated benefits of vaccination strongly motivated caregivers to immunize their children. However, sporadic vaccine stockouts and persistent health system challenges, including negative attitudes among healthcare providers, affected uptake [18, 20]. Various activities were implemented to improve caregiver knowledge and understanding of the vaccine schedule. The countries utilized local communication channels, such as community information centres and local radio stations. Social announcements were also developed in local languages, and healthcare worker job aids facilitated the education of caregivers at the immunization clinics. All three countries also reported the essential role of local health committees, opinion leaders, and community volunteers in educating and generating demand for the malaria vaccine in the communities.

In all three countries, healthcare workers were encouraged to implement malaria vaccine screening initiatives during vaccination visits, at well-child and outpatient clinics to identify and vaccinate eligible children, thereby reducing missed vaccination opportunities.

**Fig. 4:**
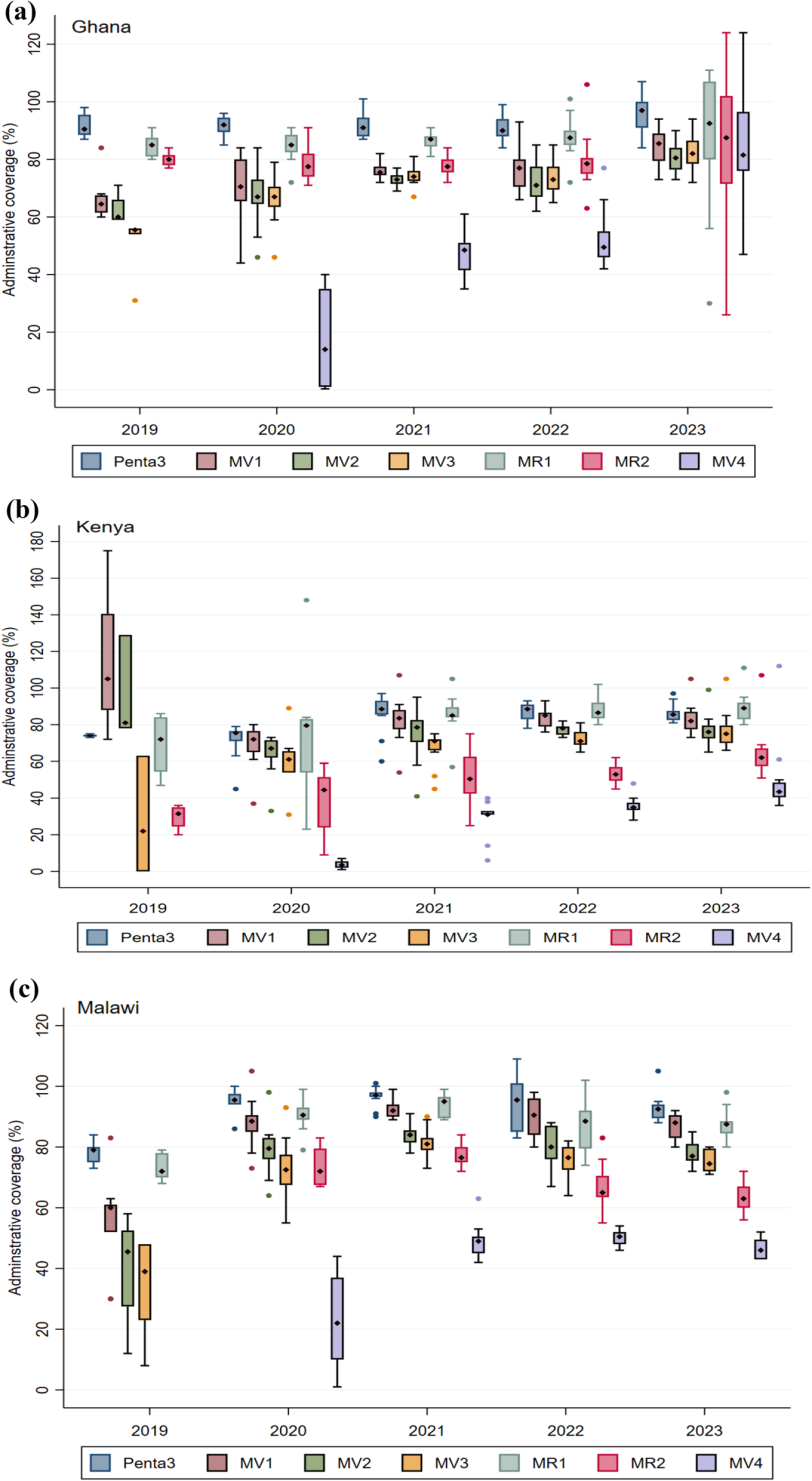
**A**–**C** Trends of coverage of the RTS, S malaria vaccine in the initial pilot areas (2019–2023) in Ghana, Kenya, and Malawi. Coverage is presented as the median with the interquartile range. The graph also includes coverage of the third dose of the Pentavalent vaccine and first and second doses of the Measles-Rubella vaccine. Vaccination coverage = (Number of vaccine doses administered)/(Number of children in the target population) X 100

Coverage of the fourth dose presented a particular challenge, and some studies elucidated key barriers to uptake, including the imprecise understanding of fourth dose eligibility and the age (timing) for the fourth dose among healthcare workers and caregivers[17, 19, 20]. While the other countries maintained the schedule, Ghana switched timing of the fourth dose from age 24 months to 18 months, to align with second dose measles-rubella vaccine (MR2), meningococcal type A (Men A) vaccine, vitamin A supplementation, ITN distribution, and growth monitoring in February 2023; this significantly improved coverage of the fourth dose (Fig. 4A).

### Data capture and management

Data collection tools were updated to record RTS,S vaccination doses, including service delivery registers, home-based immunization records (HBIR) or child health record books, tally sheets, and immunization monitoring charts. The changes were reflected in the District Health Information Management System (DHIS-2) data collection tools. In Ghana and Kenya, the RTS,S malaria vaccination data elements in the DHIS-2 platform were activated for only implementing districts as a quality control measure to prevent inadvertent reporting of RTS,S doses. This has been noted for other vaccines and health interventions. Malawi migrated malaria vaccination data from the District Vaccine Data Management Tool (DVMT-2) to the District Health Information System (DHIS). The delayed update of the ‘under two’ immunization registers in Malawi created initial challenges for healthcare workers regarding the documentation of fourth dose coverage among children who received their fourth dose after 24 months of age, potentially contributing to the low coverage. The updated recording tools improved data quality by strengthening documentation at service delivery points and facilitating data validation processes.

Strategies to identify, trace, and vaccinate eligible children who missed doses included using defaulter registers in Ghana, implementing reminder cards in Malawi, and utilizing a mother–child health handbook in Kenya. The strategies seemed to have improved coverage, except for the fourth dose, which remained relatively low.

Before updating the HBIRs in all three countries, identification and malaria vaccine schedule stickers were developed and affixed in the HBIRs for age-eligible children in the implementing areas. These proved vital in prompting healthcare workers to screen eligible children for administration of subsequent doses. During the expansion phase, Ghana leveraged existing digital infrastructure to facilitate the implementation of the electronic immunization registry (eIR) (using the e-Tracker module on the DHIS platform) in seven districts.

In reiterating the key points regarding data management, countries must ensure that existing immunization data management systems are updated early and pre-tested. Recording and monitoring tools must be updated and distributed ahead of vaccine introduction. Countries must strengthen existing defaulter tracing strategies or develop new ones. Where available, the use of digital solutions, such as digital microplanning, supportive supervision, and electronic immunization registries, can facilitate effective monitoring of vaccine coverage and defaulter tracing.

### Pharmacovigilance

Adverse events were reported through service delivery points using routine surveillance systems to the national regulatory authorities. Healthcare providers maintained permanent vaccination records and were required to report all adverse events following immunization (AEFI) and adverse events of special interest (AESI) to the next level immediately. AEFI reporting by parents or guardians was encouraged through existing channels, including call centres, the Med Safety App (in Ghana), and online reporting systems [21]. The existing systems for addressing rumours were enhanced at all levels to maintain and improve confidence in immunization services. Communications on vaccine safety-related events were managed at the subnational levels with support from the national level.

The subnational AEFI investigation teams investigated all serious AEFIs. The Technical Advisory Committee on the Safety of Vaccines and Biological Products conducted causality assessments and provided feedback to stakeholders and communities to dispel rumours and strengthen vaccine confidence. The MVIP contributed to strengthening safety surveillance for other childhood vaccines in the national schedule, and the lessons are documented in another manuscript in this collection [22].

### Monitoring and evaluation

Pre-introduction assessments were conducted in the vaccination areas, followed by a rapid post-introduction assessment at least 2 weeks after the vaccine was introduced. All three countries conducted routine and targeted monitoring through supportive supervision and performance review meetings to assess, among other things, service delivery concerning documentation of vaccine administration, calculation of administrative coverage, vaccine management, vaccine safety, acceptability, understanding of the vaccine schedule and eligibility, and assessment of quality improvement activities such as refresher training on service delivery. The NIP and NMP jointly conducted Initial supervisory visits just after vaccine launch. This joint visit was essential to ensure an accurate response to questions from healthcare workers and to align on lessons learned by the programmes. Following the initial period of joint supervision, which was limited to several months after introduction, supervision was integrated into the existing NIP activities. Partners also supported monitoring activities. In Ghana, the supportive supervision checklist was digitized, utilizing the Open Data Kit (ODK) to facilitate the efficient synthesis of findings and provide timely feedback to healthcare workers, thereby enhancing service delivery. Written feedback was shared with regional administrative levels to enable the implementation of tailored recommendations that strengthen service delivery and malaria vaccine uptake. Regular data validation and performance review meetings were conducted (monthly and quarterly) at the subnational and national levels. Participants included healthcare workers directly involved in immunization service delivery, data managers, and supervisors at the subnational levels. The performance review meetings included partners, other external stakeholders, and key community members. The meetings identified best practices and gaps to facilitate the development of improvement plans. Post-introduction evaluations (PIEs) were conducted more than a year after vaccine introduction, due to the COVID-19 pandemic. Still, the timing also provided the opportunity to evaluate performance, community perception, and uptake of the fourth dose, including the usefulness of innovative healthcare worker training tools and job aids developed during the pandemic to support uptake. Key informants, including healthcare workers and caregivers at all levels of the health system, were interviewed to document lessons for improving service delivery and uptake. Findings from the PIEs are published in a companion paper in this collection [23]. Based on these experiences, countries planning to introduce malaria vaccines should develop comprehensive monitoring and evaluation (M&E) frameworks to track progress and facilitate effective accountability.

### Communication, demand generation, and initial anti-vaccination sentiment

Some vaccine hesitancy may characterize the early phases of new vaccine introduction due to uncertainties and concerns about vaccine safety and efficacy [24]. Malaria vaccine hesitancy varied across the three countries. In Ghana, hesitancy in the initial phases of the pilot was fuelled by videos and disinformation on various social media platforms, developed mainly by anti-vaccine actors in other countries and outside of Africa [11]. Rapid and thorough response from the MoH of Ghana and other medical and scientific experts supporting the malaria vaccine mitigated the misinformation and negative rumours. Hesitancy among healthcare workers contributed to the selective administration and low coverage of the malaria vaccine in specific areas of Ghana. In all three countries, scientists involved in the pivotal Phase 3 RTS,S clinical trials and part of the MVIP evaluation consortium facilitated public education, reiterating the safety and efficacy of the vaccine. This helped build public trust during the early phase of vaccine deployment.

All three countries adapted WHO risk communication materials to develop country-specific risk communication strategies and activities as part of their malaria vaccine introduction plans. Activities included malaria vaccine spokesperson training, media education, targeted community engagement to address misunderstandings or questions, regular monitoring of misinformation and rumors (including supportive supervision activities), and response scenarios. Working through the TWG, the MoH of Ghana trained and designated communication focal points for each vaccinating district to engage community-level stakeholders and address rumours rapidly. Additionally, separate workshops were organized for senior editors and journalists to facilitate the dissemination of accurate information on the malaria vaccine. Social media was leveraged to disseminate accurate information through infographics and short messages.

In Kenya, spokesperson training was conducted for selected healthcare staff at the national, county, and sub-county levels to enhance their capacity to address the public and media on malaria vaccine-related issues. The trainings targeted cadres including directors, public health officers, health promotion officers, immunization and malaria programme staff, and community health focal points. In Malawi, various cadres of spokespersons were trained, including journalists, health promotion officers, NIP officers, and malaria control programme officers at both national and district levels. In addition, interactive health education sessions were conducted for caregivers.

In all the countries, editorials and commentaries from medical professionals and community leaders were published periodically, particularly at key advocacy opportunities such as World Malaria Day and African Vaccination Week, to disseminate evidence on safety, impact, and malaria vaccination as part of recommended malaria prevention measures. These interventions were crucial in addressing rumors and anti-vaccine sentiments, contributing to an improved uptake of the malaria vaccine. However, in Malawi, the initiative to use caregivers as peer influencers to generate demand for the malaria vaccine was less effective, as no significant improvement in malaria vaccine coverage was observed following the intervention. The suboptimal impact of the initiative was potentially due to inadequate resources to support the peer influencers and competing demands on their time, given their roles as primary caregivers.

Vaccine hesitancy was not widespread in any country but limited to specific areas. Timely mitigation measures and interventions, anchored in robust risk communication plans, facilitated an effective response to rumours, misinformation, and disinformation, resulting in a limited impact of vaccine hesitancy on vaccine uptake.

### Trends of coverage and dropout rate

Malaria vaccine coverage data were summarized from service delivery registers at the facility level and entered into the respective health management information system (HMIS) platforms. Vaccine coverage was estimated using the same approach and population denominators as for other childhood vaccines. This was, therefore, subject to inherent data quality issues. Interventions to improve data quality for estimating vaccine coverage were not limited to malaria vaccines but also extended to the broader health system. Regarding population denominators, all three countries revised their target populations for childhood vaccines, including malaria vaccines, using data from respective national censuses conducted during the pilot, in line with national policies.

A total of 6,575,934 RTS,S malaria vaccine doses were administered between the countries over the pilot period. Approximately 39% (2,569,752), 33% (2,189,633), and 28% (1,816,549) of the doses were delivered in Ghana, Kenya, and Malawi, respectively. Vaccination coverage improved steadily over the pilot period as observed in the initial implementing areas (Fig. 4A–C) [25]. First-dose (MV1) coverage reached at least 80% by the end of the pilot, with a substantial improvement in coverage of three doses, reaching at least 75% in the three pilot countries. Coverage of the fourth dose (MV4) was comparatively lower, averaging around 46%, but significant improvements were noted in Ghana after the schedule change. The fourth dose coverage increased from 14% in 2020 to 49% in 2021, 50% in 2022, and then rose substantially to 82% in 2023. A similar pattern was observed for Kenya, although less pronounced than in Ghana, with a rise from 4% in 2020 to 31% in 2021, 35% in 2022, and 44% in 2023. In Malawi, the steep rise in fourth dose coverage from 22% in 2020 to 49% in 2021 was followed by a marginal increase to 51% in 2022, before declining to 46% in 2023. The sharp increase in fourth dose coverage in Ghana was due to alignment of the scheduled age with the 18-month vaccination time point. It is instructive to note that the vaccines (MR2 and MenA) administered at 18 months of age had relatively high coverage (around 80%), contributing to the observed increase in the fourth dose coverage in Ghana. Other activities implemented to optimize fourth dose coverage included a catch-up vaccination campaign and periodic intensification of routine immunization (PIRI) activities. The median coverage of the fourth dose remained around 80%, suggesting that aligning with 2YL vaccines, which already had high coverage, was the most critical factor in achieving this level of coverage.

**Fig. 5:**
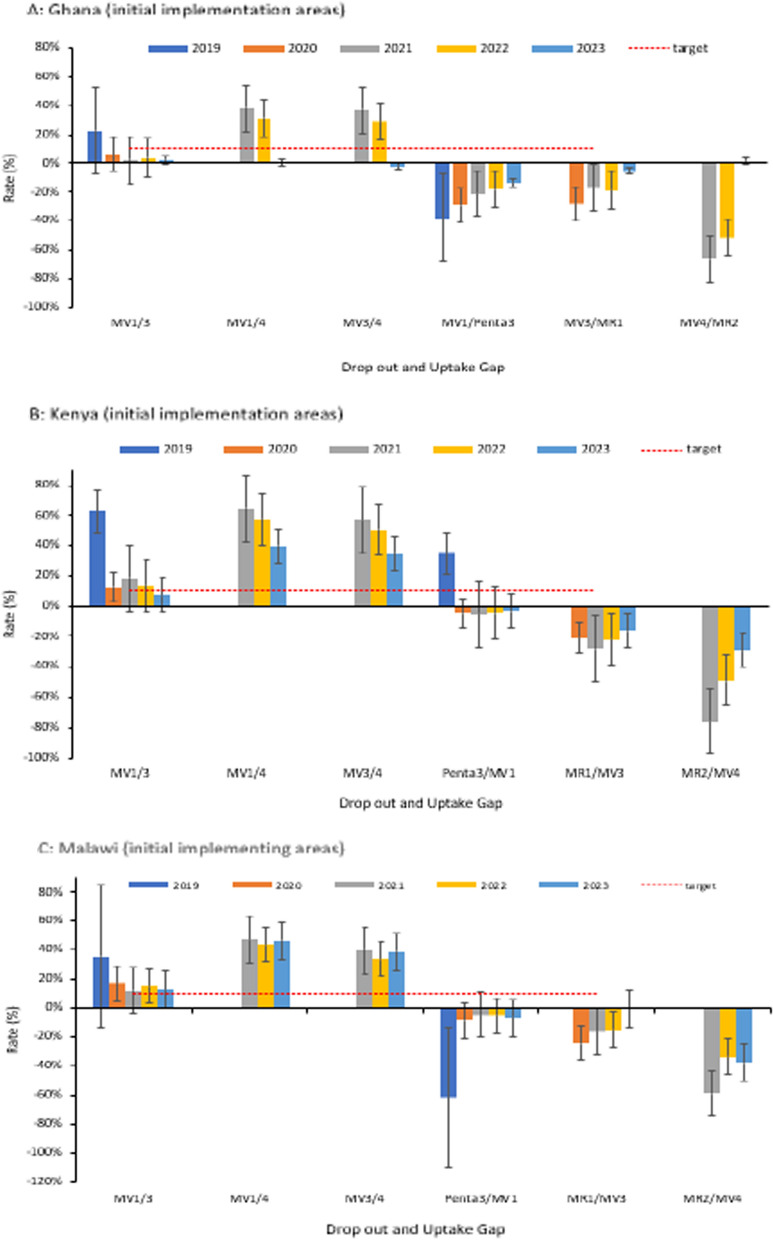
**A**–**C** Trend of RTS,S malaria vaccine dropout rate and uptake gap in the initial implementing areas; 2019–2023. The drop-out rate measures service utilization and the retention of children in completing the scheduled series of a particular vaccine. Rates below 10% are generally considered optimal service utilization. The uptake gap compares vaccines that are provided around or at the same age to assess missed opportunities for vaccination. The negative rates for the uptake gaps show lower coverage of the malaria vaccine compared to vaccines provided at a comparable age. No negative drop-out rates are reported in the Figure. Dropout rate = (Number of doses administered for an initial dose−Number of doses administered for a later dose)/(Number of doses administered for an initial dose) X 100; Coverage gap = (Number of doses administered for target vaccine (RTS,S)−Number of doses administered for comparator vaccine)/(Number of doses administered for target vaccine (RTS,S) X 100

In the initial pilot areas, the dropout rates and coverage gap for the fourth dose, improved over time (Fig. 5A–C). Dropout rates between the first and third dose (MV1/3) were lower or slightly above the prescribed target of 10%. There were gaps between malaria vaccine coverage and vaccines given at comparable ages. For example, coverage gaps between the third dose (MV3) and MR1 suggest a missed opportunity for screening children who reported for MR1 vaccination. The scheduled visits for Penta3 and MR2 could also have been utilized to provide reminders to caregivers.

The initial uptake patterns of the malaria vaccine within the first year of introduction (Supplement Figs S3A-S3F) varied between the respective countries, related mainly to the vaccine introduction launch approach as outlined previously, and other contextual factors documented in studies from the respective countries [17–19, 26, 27]. Given the schedule of the fourth dose, uptake was expected to begin in the second year of vaccine introduction in the expansion areas. The MV4 coverage recorded in the expansion areas during the first year of implementation may have been due to an influx of children from neighbouring communities in the initial implementing areas who had already received four doses (Supplement Fig. S2). On the other hand, data recording errors could have contributed to the observation [28].

In addition to strengthening defaulter tracing mechanisms to identify and vaccinate missed children, efforts to increase MV4 coverage through periodic intensification of routine immunization (PIRI) activities yielded modest outcomes. The PIRIs were particularly important following the disruption of service delivery during the COVID-19 pandemic, which resulted in malaria vaccine stockouts due to global supply chain constraints. Malaria vaccine catch-up activities in Ghana were integrated with reactive and preventive mass campaigns such as COVID-19 vaccination and seasonal malaria chemoprevention (SMC).

Regarding defaulter tracing strategies, in Ghana, the newly introduced defaulter tracing register was used to document and track children who missed their scheduled vaccine doses, including those for malaria. These registers were used to target home visits by community healthcare workers, and were also used by community health volunteers to identify children during outreach immunization sessions. In Kenya, extensive outreach sessions and community volunteers facilitated the tracing of defaulters, and in Malawi, reminder cards were issued to caregivers. Community peer educators in Malawi also linked eligible children to healthcare workers, which helped improve coverage and reduce the dropout rate among children. These activities generally improved defaulter tracing but also required sustained funding to ensure effectiveness.

### Programme integration and continued adherence to other malaria interventions

The MVIP successfully introduced the malaria vaccine into the childhood immunization programme without disrupting the uptake of other childhood vaccines, malaria prevention measures, and health-seeking behaviour [2]. In all three pilot countries, this was achieved, in part, through structured stakeholder engagement at both the national and subnational levels across the two programmes.

Technical discussions with malaria and immunization partners, as well as health management teams at national, district, and sub-district levels, were critical for disseminating accurate information about the introduction of malaria vaccines. Joint participation of the two programmes in sub-national trainings was considered essential to ensure alignment of the programmes on the use of malaria vaccines and other malaria control interventions and to emphasize the complementarity of existing malaria control interventions, which include the malaria vaccine. Supervisory visits and community education reinforced the messaging on the continued use of all malaria prevention tools, including sleeping under insecticide-treated bed nets every night and seeking prompt care for the diagnosis and treatment of febrile illnesses. Independent assessments conducted during the evaluation revealed that introducing the malaria vaccine did not adversely affect the uptake of other malaria interventions and childhood vaccines [2].

## Summary

The malaria vaccination programme is a cross-cutting intervention; therefore, efforts must be made to optimize collaboration between key stakeholders, including national immunization and malaria control programmes. Collaboration is necessary, for example, in identifying subnational areas according to local disease burden to guide vaccine deployment and optimize its public health impact. The MoHs established coordination mechanisms in the pilot countries, including technical working groups that brought together experts from the two programmes and other relevant agencies to facilitate the vaccine introduction. This promoted accountability and information sharing to mitigate potential conflicts that could have arisen due to suboptimal communication.

Given that healthcare workers are regarded as the face of the healthcare system, their stance on immunization has far-reaching consequences for community acceptance. In addition to targeting caregivers with demand generation messages, it is essential to strengthen healthcare worker education, focusing on all cadres to foster support for the introduction of malaria vaccines. This will facilitate an understanding of vaccine safety and eligibility, thereby reducing missed vaccination opportunities and enhancing coverage.

Adopting a context-based approach to programme launch is critical, and a trade-off between high- and low-profile strategies may be considered. Although a high-profile launch may rapidly achieve high coverage, the potential arousal of anti-vaccine sentiments should be anticipated and mitigated. On the other hand, a low-profile approach may limit the spread of anti-vaccine sentiments, but creative and locally tailored solutions are needed to enhance the rate of vaccine uptake. Regardless of the approach, it is vital to ensure early and continuous engagement of caregivers and community members to improve and sustain uptake.

The four-dose schedule of the malaria vaccine presents both opportunities and challenges, depending on the extent to which countries find a balance between aligning the schedule with existing child health interventions. The multiple service contact points associated with introducing the malaria vaccine should be leveraged to improve the delivery of other child health interventions. Programmatic feasibility is key, such as aligning the fourth dose with other second-year-of-life vaccines for which there is already good coverage.

Data collection tools should be adapted to record all administered doses, and data quality mechanisms must be accurately established to ensure the timely and effective use of evidence-based decision-making by frontline healthcare workers and health authorities. Recording and monitoring tools should be updated early (at least 6 months before introduction), pre-tested, and distributed to health facilities before vaccinations commence.

The MVIP achieved key successes, including introducing the malaria vaccine as part of the childhood immunization programmes; strengthening routine vaccine pharmacovigilance; and continued adherence to other malaria interventions, which were attributed to the strong collaboration between the NIP and NMP. Inadequate understanding of age eligibility criteria in the early phase of introduction and low fourth dose coverage were among the key challenges encountered by the pilot countries. Lessons from this pilot, including those on introduction during the COVID-19 pandemic, should be leveraged to strengthen health system resilience in delivering vaccines and maximizing the impact on malaria vaccine uptake in future introductions.

## Conclusions

This paper summarizes the experiences and lessons from the pilot introduction of malaria vaccine in Ghana, Kenya, and Malawi. Consistent with observations regarding uptake of other new vaccines, coverage improved and stabilized over time. The pilot highlighted key lessons (Table 1) for future malaria vaccine introductions: (1) clearly outlined roles and responsibilities of key stakeholders including NIP and NMP for effective collaboration; (2) appropriate approach to vaccine introduction launch, communication, and demand generation to enhance vaccine uptake; (3) flexibility with dose scheduling to optimize coverage; and (4) updated data collection tools for accurate documentation and data quality.

**Table 1:** Key recommendations for new vaccine introductions based on MVIP experience

WHO Health System component	Programmatic areas	Key components for future vaccine introductions
Leadership/Governance	Programme coordination	Establish national and subnational coordination mechanisms early to ensure smooth rollout and adequate lead time to address bottlenecks Establish malaria vaccine technical working groups and subcommittees Ensure optimal participation from national malaria and immunization programmes Map funding gaps, identify potential partners, and mobilize additional resources to address them
Service delivery	Communication, Advocacy, and Social Mobilization	Use data and information to understand the community context Early stakeholder mapping and engagement to build champions and mitigate risks Ensure health messages effectively communicate the importance of vaccination across the four-dose schedule, tailoring them for healthcare workers, caregivers, and other stakeholders Develop and communicate clear messaging on vaccine benefits and use alongside other malaria prevention interventions Leverage social media to strengthen public education using short messages and infographics Monitor and evaluate communication activities and course correct as needed
Service delivery	Service delivery and training	Prioritize targeted strategies and selection criteria based on local epidemiological data and programmatic considerations Vaccination schedule should be flexible: The use of an expanded age approach for the first dose is encouraged, but this should be accompanied by monitoring of vaccine stock Align dose schedules, especially the fourth dose, with other vaccines and interventions in the child health package Implement catch-up vaccination aligned with national policies to ensure maximum coverage of the at-risk population Develop handy job aids on vaccine eligibility criteria for healthcare workers Strengthen or develop innovative strategies for defaulter identification and tracking Integrate with other immunization activities (e.g., training, supply chain, data management) and other malaria prevention (e.g., ITN, PMC, and SMC) and child health interventions
Health information system	Data management	Incorporate data quality improvement into vaccine introduction plans Develop, revise, and pre-test recording and monitoring tools early Train healthcare workers on updated data recording and monitoring tools Leverage electronic immunization registries (as demonstrated by the COVID-19 vaccination), where available
Health Information Systems	Monitoring and evaluation	Establish comprehensive monitoring and evaluation (M&E) frameworks to strengthen accountability Conduct rapid-post introduction monitoring to identify early implementation challenges Plan for post-introduction evaluation (allowing enough time to evaluate fourth dose uptake) Invest in support supervision and continuous monitoring to enhance uptake as part of an integrated approach Consider digital supervisory tools and other digital solutions where available and feasible (e.g., ODK) for support supervision
Health Information Systems Access to Essential Medicines	Pharmacovigilance	Strengthen adverse event reporting using different channels, for example, the Med Safety App, call centre, and online reporting

## Supplementary Information

Supplementary material 1.

## Data Availability

No datasets were generated or analysed during the current study.

## Abbreviations

ACSM: Advocacy, communication, and social mobilization
AEFI: Adverse event following immunization
AESI: Adverse event of special interest
CIC: Community Information Centre
CSO: Civil Society Organization
DHIMS: District Health Information Management System
DHIS: District Health Information System
DTP: Diphtheria-tetanus-pertussis
DVMT: District vaccine data management tool
eIR: Electronic Immunization Registry
EMA: European Medicines Agency
GAS: Ghana Academy of Arts and Sciences
HBIR: Home-based Immunization Records
HMIS: Health Information Management System
ITN: Insecticide-treated bed net
MoH: Ministry of Health
MVIP: Malaria Vaccine Implementation Programme
MenA: Meningococcal Type A
MR: Measles rubella
MV: Malaria vaccine
NITAG: National Immunization Technical Advisory Group
NIP: National Immunization Programme
NMP: National Malaria Programme
NRA: National Regulatory Authority
ODK: Open data kit
PIE: Post-introduction evaluation
PIRI: Periodic intensification of routine immunization
PMC: Perennial malaria chemoprevention
PPB: Pharmacy and Poisons Board
TWG: Technical Working Group
SMC: Seasonal Malaria Chemoprevention
WHO: World Health Organization
2YL: Second Year of Life
