# Supplementary material for: Subnational introduction of the RTS,S/AS01E malaria vaccine into routine immunization: experience and lessons from the three pilot countries
Source: Malar J. 2025 Jul 28;24:244. doi: 10.1186/s12936-025-05484-6 (PMC12305954; doi:10.1186/s12936-025-05484-6)
Supplement: Supplementary file 1 — Supplementary material 1. [file 12936_2025_5484_MOESM1_ESM.docx]

**Supplementary material**

*Subnational introduction of the RTS,S/AS01E malaria vaccine into routine immunization: Experience and lessons from the three pilot countries.*

Rose J’alango, Kwame Amponsa-Achiano, Mike Chisema, Keziah Malm, Lydiah Khalayi, Brenda Mhone, Wahjib Mohammed, Franklin Asiedu-Bekoe, Adam Haji, Josephine Njoroge, Boston Zimba, Esther Chirwa, Peter O Tweneboah, Jackson Sillah, Mgaywa GMD Magafu, Cynthia Bergstrom, Tracey Goodman, Jenny Walldorf, Kristen Kelleher, Eliane Pellaux-Furrer, Mary J Hamel, Michael R Adjei, Rafiq NA Okine


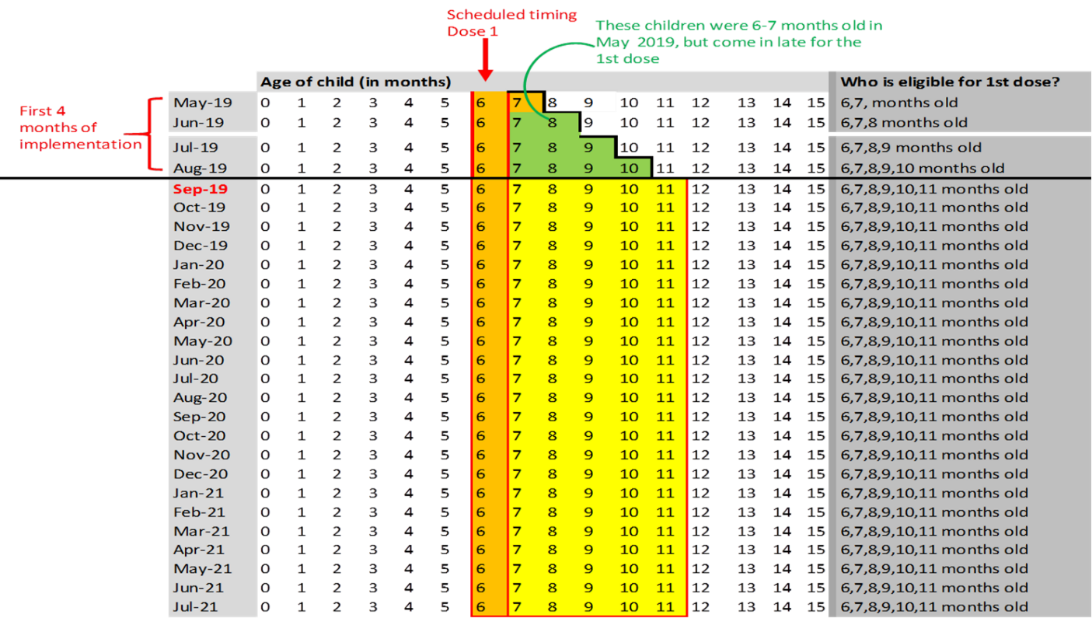


**Figure S1:** RTS,S malaria vaccination eligibility criteria, MVIP, Ghana; 2019-2023. The job aid shows the age eligibility for the first dose of the RTS,S vaccine in Ghana. In the first month of introduction, children aged 6 and 7 months were eligible for the first dose (orange shaded area). The green-shaded area indicates the catch-up of children who missed the initial introduction. The scheduled age for the first dose (6 months) is highlighted in orange. The dose 1 eligibility was further extended to include all children aged 6-11 months (yellow shaded areas).


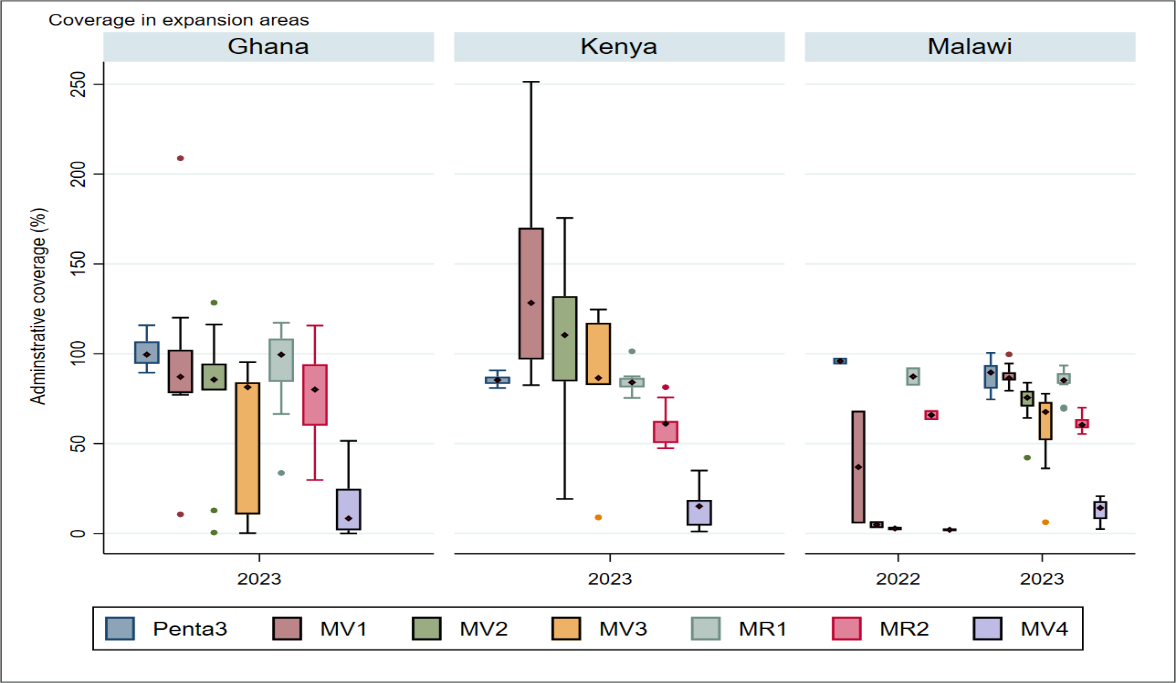


**Figure S2.** Coverage of RTS,S malaria vaccine in the expansion pilot areas, MVIP (2023)

**Table S1.** Summarized coverages, initial implementing areas, 2020-2023

| Vaccine | Ghana | | | | | Kenya | | | | | Malawi | | | | |
| --- | --- | --- | --- | --- | --- | --- | --- | --- | --- | --- | --- | --- | --- | --- | --- |
|  | Coverage, median (IQR) | | | | | Coverage, median (IQR) | | | | | Coverage, median (IQR) | | | | |
|  | 2019 | 2020 | 2021 | 2022 | 2023 | 2019 | 2020 | 2021 | 2022 | 2023 | 2019 | 2020 | 2021 | 2022 | 2023 |
| Penta3 | 91 (89,96) | 92 (90,95) | 91 (88,95) | 90 (88,94) | 97 (91,100) | 74 (74,75) | 76 (71,77) | 89 (86,93) | 89 (83,91) | 86 (83,88) | 79 (75,80) | 96 (94,98) | 97 (97,98) | 96 (85,101) | 93 (90,94) |
| MV1 | 65 (62,68) | 71 (66,80) | 76 (75,78) | 77 (71,80) | 86  (80,89) | 105 (88,141) | 72 (65,77) | 84 (78,88) | 85 (79,87) | 82 (78,87) | 60 (52,61) | 89 (85,91) | 92 (90,94) | 91 (84,96) | 88 (83,91) |
| MV2 | 60 (59,66) | 67 (65,73) | 73 (72,75) | 71 (67,78) | 81 (77,84) | 81 (78,129) | 67 (62,72) | 79 (71,83) | 78 (75,79) | 76 (73,80) | 46 (28,53) | 80 (76,83) | 84 (82,86) | 80 (76,87) | 77 (76,81) |
| MV3 | 56 (54,56) | 67 (64,71) | 74 (73,76) | 73 (70,78) | 82  (79,87) | 22  (0,63) | 61 (54,66) | 71 (66,72) | 71 (69,76) | 75 (70,80) | 39 (23,48) | 73 (68,78) | 81 (79,83) | 77 (73,80) | 75 (72,80) |
| MR1 | 85 (81,88) | 85 (83,89) | 87 (85,88) | 88 (85,90) | 93 (80,107) | 72 (55,84) | 80 (54,83) | 85 (84,90) | 87 (84,92) | 89 (83,92) | 72 (70,78) | 91 (89,93) | 95 (90,97) | 89 (80,92) | 88 (85,89) |
| MR2 | 80 (78,82) | 78 (74,82) | 78 (76,80) | 79 (75,81) | 88 (72,102) | 32 (25,35) | 45 (24,52) | 51 (43,63) | 53 (50,57) | 62 (58,67) |  | 72 (68,80) | 77 (75,80) | 65 (64,71) | 63 (60,67) |
| MV4 |  | 14 (1,35) | 49 (42,51) | 50 (46,55) | 82 (76,97) |  | 4  (2,6) | 31 (31,33) | 35 (34,38) | 44 (41,49) |  | 22 (10,37) | 49 (45,51) | 51 (48,52) | 46 (43,50) |


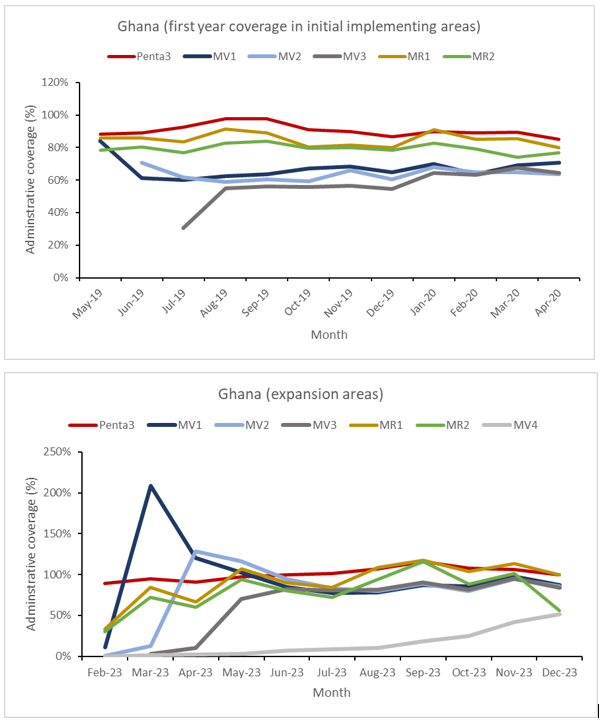


**Figures S3A-B.** Ghana**:** Trends of coverage by month in the first years of pilot, Initial versus Expansion areas, MVIP; 2019-2023


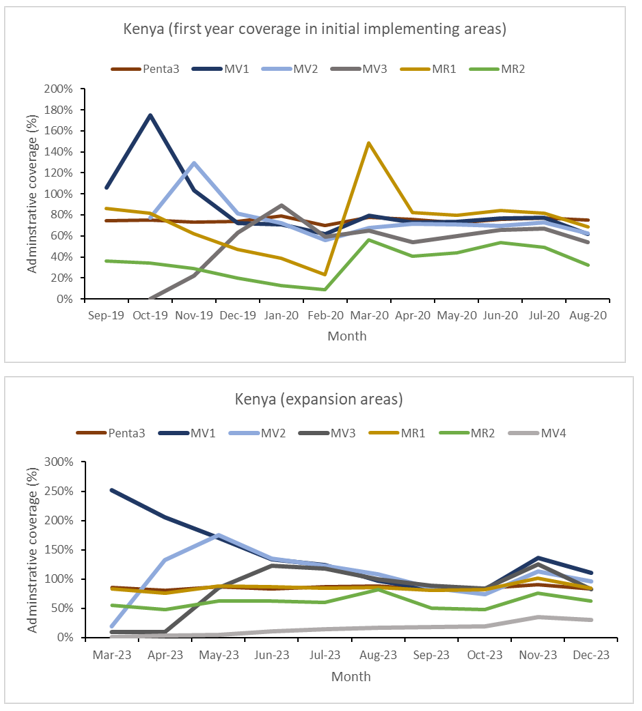


**Figures S3C-D. Kenya:** Trends of coverage by month in the first years of pilot, Initial versus Expansion areas, MVIP; 2019-2023


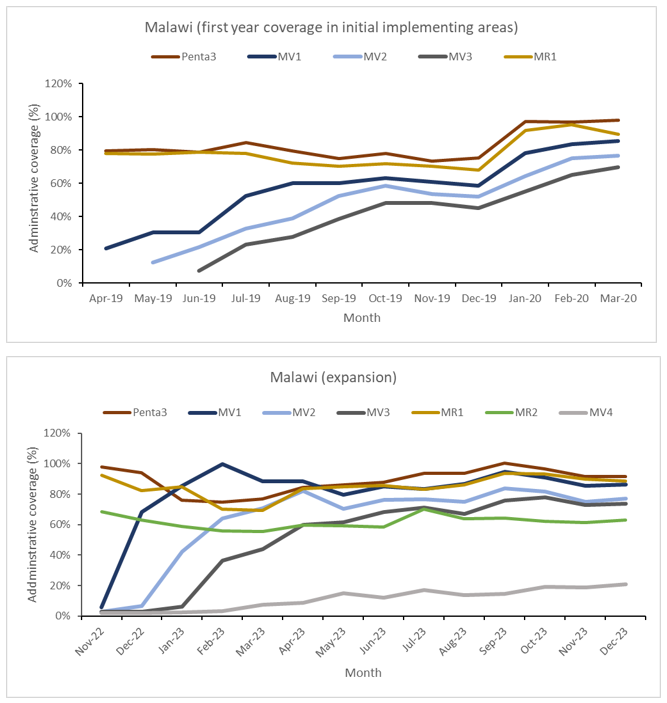


**Figures S3E-F.** Malawi**:** Trends of coverage by month in the first years of pilot, Initial versus Expansion areas, MVIP; 2019-2023
